# Diagnostic Imaging of Diseases Affecting the Guttural Pouch

**DOI:** 10.3390/vetsci10080525

**Published:** 2023-08-16

**Authors:** Aurélie Thomas-Cancian, Emilie Ségard-Weisse, Bianca Drumond, Jean-Luc Cadoré

**Affiliations:** 1 Pôle de Compétences en Santé Équine, Ecole Nationale Vétérinaire de Lyon, VetAgro Sup, Université de Lyon, F-69280 Marcy l’Etoile, France; emilie.segard@vetagro-sup.fr (E.S.-W.); bianca.drumond@vetagro-sup.fr (B.D.); jean-luc.cadore@vetagro-sup.fr (J.-L.C.); 2 UMR 754 INRAE «Infections Virales et Pathologie Comparée», Université de Lyon, F-69003 Lyon, France

**Keywords:** guttural pouch, radiography, computed tomography, horse

## Abstract

**Simple Summary:**

The guttural pouch of the horse is a diverticulum of the auditory tube and has a complex anatomical structure. Disease of the guttural pouch can lead to neurological signs and hemorrhage due to its close relation to major vessels and some cranial nerves. Endoscopy allows direct visualization of the pouch and is considered to be the gold standard for its evaluation. Nevertheless, diagnostic imaging can bring useful additional information and this review article describes the value of each technique and the main imaging findings to be expected in the diagnosis of guttural pouch disease.

**Abstract:**

The most common diseases of the guttural pouch are empyema, tympany, mycosis and temporohyoid osteoarthropathy. The challenge in diagnosis of guttural pouch diseases lies in the complex anatomy of the guttural pouch and adjacent associated structures. Diagnostic imaging is a good complement to endoscopy for the diagnosis of some guttural pouch diseases, especially to make a full assessment of the lesions involving the pouch and surrounding structures. This review article describes the value of each diagnostic imaging technique in the diagnosis of guttural pouch disease and the corresponding imaging findings. Radiography is generally used as the first line to complement endoscopic findings, and can give useful additional information although it is limited by superimposition. Ultrasonographic examination of the guttural pouch is of limited value due to the presence of gas in the guttural pouch but can eventually be used to detect fluid within the pouch or can help to evaluate the soft tissues located lateral and ventral to the guttural pouch. Cross-sectional imaging, especially CT, is increasingly available and appears to be the best technique to fully assess the surrounding soft tissues and to precisely identify lesions of the temporohyoid apparatus, temporal bone and skull base that are associated with guttural pouch disease.

## 1. Introduction

The guttural pouch is a diverticulum of the auditory tube that connects the pharynx to the middle ear bilaterally. This diverticulum has a complex anatomical structure, consisting of a cavity divided by the stylohyoid bone into a small lateral recess and a larger medial recess [1]. The guttural pouch walls are in close relation to large vessels (maxillary vessels and external and internal carotid arteries) and some cranial nerves (VII, IX, X, XI and XII) [1]. Conditions affecting the guttural pouch may consequently contribute to severe hemorrhage and neurological dysfunction, which can be fatal [2,3]. Adjacent medial and lateral retropharyngeal lymph nodes are also associated with some diseases of the guttural pouch and there is also intimate contact of the walls of the guttural pouch with several osseous and muscular structures, including the temporohyoid apparatus and the skull base [3,4]. Endoscopy is generally used to diagnose guttural pouch disease because it allows direct visualization of the majority of the structures of interest [3,4]. Diagnostic imaging is generally used to complement endoscopy, and can provide relevant additional information. In particular, diagnostic imaging allows for the evaluation of the structures surrounding the pouches which cannot be fully assessed with endoscopy [3,4]. This review article describes the usefulness of various imaging modalities in the diagnosis of diseases affecting the guttural pouches, and the main imaging features that can be expected for each condition.

## 2. Use and Interest of the Different Imaging Techniques for Assessing the Guttural Pouches and Associated Structures

### 2.1. Radiography

**Figure 1:**
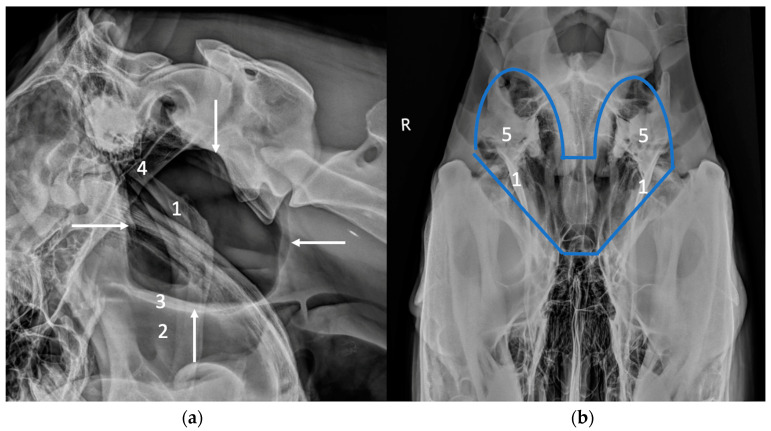
Latero-lateral (**a**) and ventro-dorsal views (**b**) illustrating the normal radiographic appearance of the guttural pouches, delineated by white arrows in (**a**) and by a blue line in (**b**). The guttural pouches are less visible in the ventro-dorsal view due to surperimposition with several bony structures. (1) Stylohyoid bones, (2) pharynx, (3) pharyngeal roof, (4) basioccipital and basisphenoid bones, (5) petrous part of the temporal bone.

Radiography is generally the first-line imaging modality to be used to complement endoscopy for the diagnosis of guttural pouch diseases (Figure 1). Air-filled guttural pouches provide a very good contrast for radiographic imaging. A lateral view centered between the ear base and the angle of the mandible is the most informative and is fairly easy to perform [5]. If necessary, both right and left lateral views or lateral views taken with slight rostro-caudal obliquity to separate left and right sides can be performed to allow lateralization of the lesion [5,6]. The radiographic examination can be completed by a ventro-dorsal (or dorso-ventral) view [5]. This view is of limited interest for the evaluation of the guttural pouch itself due to a large amount of superimposition with bony structures, but it can be useful and must be performed to assess the temporohyoid apparatus [6]. In this case, it must be perfectly positioned to be able to detect changes in the symmetry of the temporohyoid apparatus [6]. In a normal horse, the guttural pouches appear as large radiolucent structures, with a dorsal wall in contact with the skull base, and a ventral border delineating the roof of the pharynx on the lateral view [5]. They normally extend caudally up to the level of the dens of the axis [5]. The caudal contour of the pouches appears generally as a double line on the lateral view since the two pouches are rarely perfectly superimposed [5]. Detection of guttural pouch disease on radiographs is based on the visualization of changes of opacity or mass effect within the pouches, changes in their size, and modification of their contours due to adjacent structures [5].

### 2.2. Ultrasound

**Figure 2:**
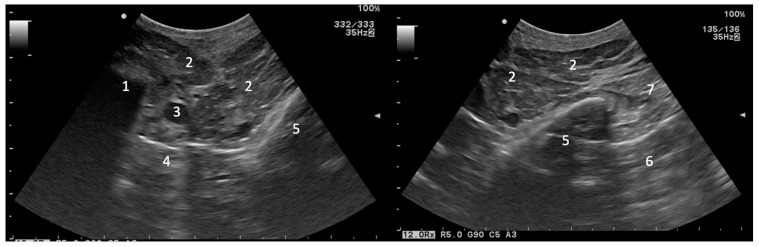
Transverse ultrasonographic images (Aloka Alpha 7, microconvex probe 5–10 MHz) of the area of Viborg’s triangle caudal to the vertical ramus of the mandible. Cranial is to the left. (1) Caudal border of the mandible, (2) parotid gland, (3) external carotid artery, (4) lateral recess of the guttural pouch, (5) dorsal extremity of the stylohyoid bone, (6) medial recess of the guttural pouch, (7) muscles lying lateral to the guttural pouch (digastric and occipitohyoid muscles).

Ultrasonography of the region of the guttural pouches is of limited value due to the presence of air in the guttural pouch and the vertical ramus of the mandible, and the consequent inability to image through them [6]. Due to their content, only the surface of normal guttural pouches can be identified in the area of Viborg’s triangle, caudally to the ramus of the mandible and deep to the parotid gland and digastric muscle, as a hyperechoic line with a dirt acoustic shadow (Figure 2) [7]. They can be seen entirely only in pathological conditions if they are filled with fluid [7]. In very specific circumstances, ultrasonography can help to localize a foreign body within the pouch, or to detect fluid within the pouch if an endoscopy or a radiography cannot be performed [7]. Ultrasonography can also be used to assess the soft tissues lateral and ventral to the pouch and may be useful in detecting lymph node involvement [4].

### 2.3. Cross-Sectional Imaging

**Figure 3:**
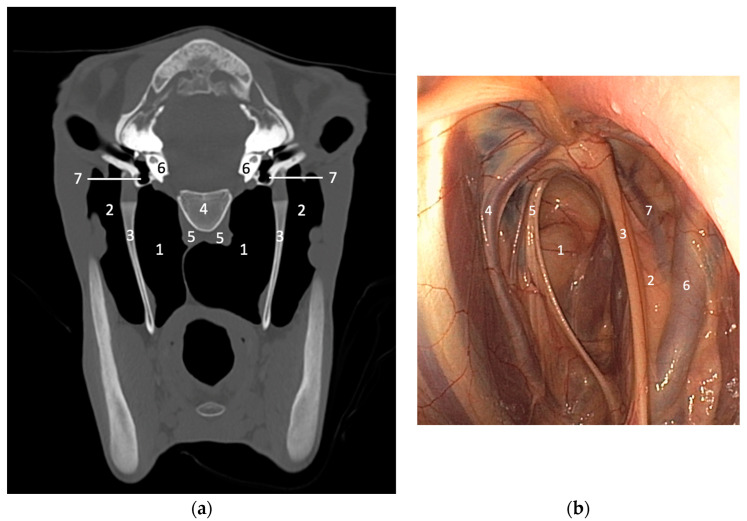
(**a**) CT transverse image (bone window) of a normal young horse at the level of the inner ear. Right is to the left. The guttural pouches and related structures can be clearly identified and evaluated without superimposition. (1) Medial compartment of the guttural pouch, (2) lateral compartment of the guttural pouch, (3) stylohyoid bone, (4) basioccipital bone, (5) rectus capitis and longus capitis muscles, (6) petrous part of the temporal bone, (7) tympanic bullae. Image courtesy of Dr Mickaël Robert. (**b**) Endoscopy of the guttural pouch of a normal horse for comparison (medial is to the left). The inside of the guttural pouch can be observed directly but the evaluation of some related structures remains incomplete compared to CT. (1) Medial compartment of the guttural pouch, (2) lateral compartment of the guttural pouch, (3) stylohyoid bone, (4) external carotid artery, (5) cranial nerves IX, X, XI and XII, (6) maxillary artery, (7) internal carotid artery.

Cross-sectional imaging, especially CT, is becoming increasingly available for imaging horse heads, particularly with the development of standing CT, allowing the elimination of general anesthesia and reduced costs [8]. The major interest of CT is due to its ability to image the complex structure of the head without superimposition [6,8]. CT also has increased soft tissue contrast compared to conventional imaging. Consequently, CT permits a better understanding of the anatomy of the guttural pouches and associated structures (Figure 3). For the evaluation of guttural pouches, CT is of particular interest to diagnose and better assess associated bony lesions, like temporohyoid osteoarthropathy, temporal bone fractures, stylohyoid bone fractures and avulsion fractures of the skull base [3,6,8,9]. MRI may be also helpful in selected cases of temporohyoid osteoarthropathy [8], and in some cases of masses involving the guttural pouches, MRI can also provide useful information about the extent of soft tissue involvement [4], but it remains less efficient than CT for evaluation of bony structures and requires general anesthesia.

## 3. Imaging Features of Diseases Affecting the Guttural Pouch

### 3.1. Guttural Pouch Empyema

**Figure 4:**
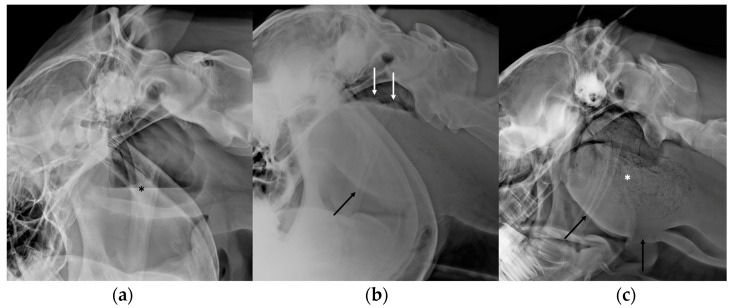
Latero-lateral views of three different horses with guttural pouch empyema illustrating the variable appearance and quantity of the material filling the guttural pouch. In (**a**), only a small amount of homogeneous fluid delineated dorsally by a gas/fluid interface (black asterisk) is visible in the ventral part of the guttural pouch. In (**b**), the pus is thicker with a convex dorsal border (white arrows) and almost completely fills the guttural pouch. In (**c**), the guttural pouch is completely filled with heterogenous thick pus with a mottled appearance (white asterisk), creating a mass effect. In (**b**,**c**) the roof of the pharynx is displaced by the filled guttural pouch (black arrows), inducing a reduction of the lumen of the pharynx and the larynx, causing respiratory distress.

**Figure 5:**
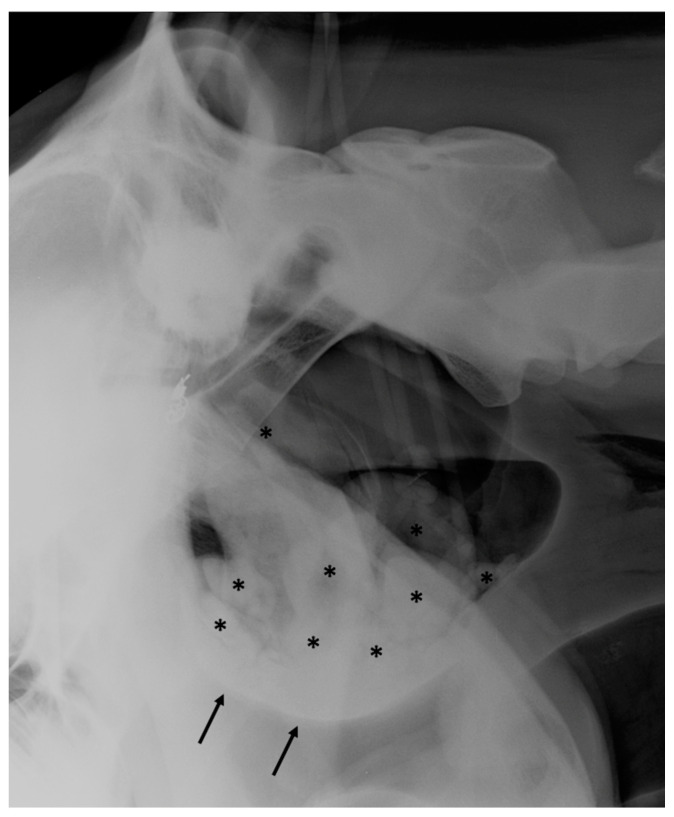
Latero-lateral view of a horse with chronic guttural pouch empyema. Several radio-opaque nodules of various size (black asterisks) are filling the guttural pouch, creating a mass effect displacing the roof of the pharynx ventrally (black arrows). A metal opaque coil can be observed along the cranial border of the guttural pouch secondary to a previous surgical embolization of the internal carotid artery.

**Figure 6:**
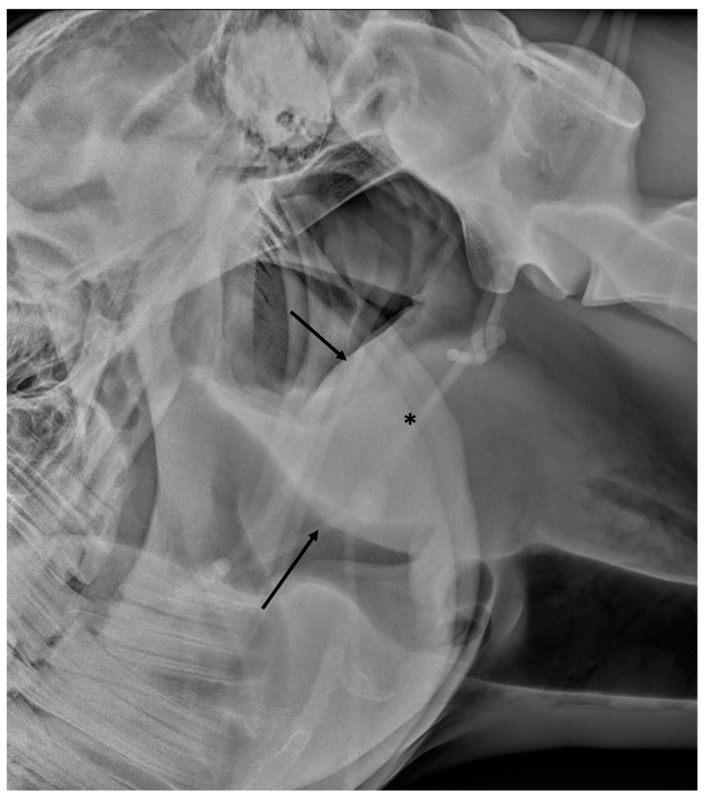
Latero-lateral view of a horse with guttural pouch empyema and severe retropharyngeal abscessation. Note the severe mass effect caused by the severely enlarged retropharyngeal lymph nodes (black asterisk), displacing the caudal border of the guttural pouch and the roof of the pharynx (black arrows).

**Figure 7:**
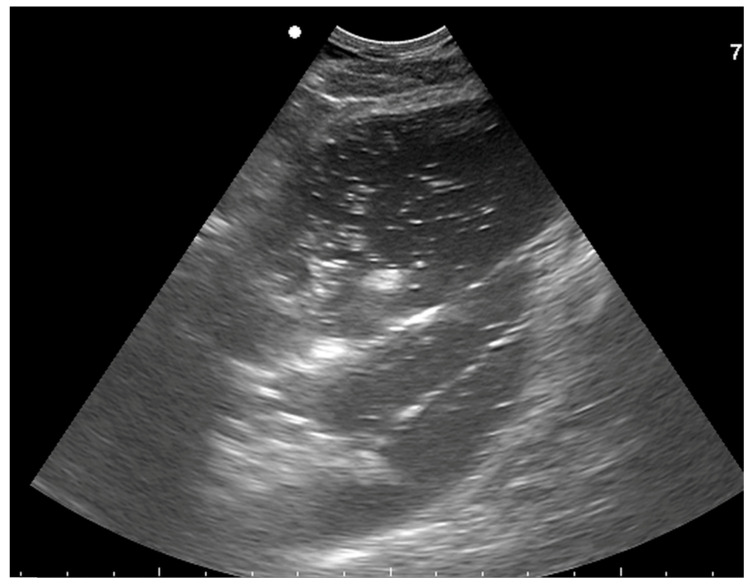
Transverse ultrasonographic image (Aloka Alpha 7, microconvex probe 5–10 MHz) of the pharyngeal area of a horse with guttural pouch empyema showing the guttural pouch markedly dilated and filled with heterogenous hypoechoic fluid. Cranial is to the left.

Empyema is the most common disease of the guttural pouch and can be caused by upper airway infection or drainage of abscesses of the retropharyngeal lymph nodes into the ipsilateral pouch [2,3,8,10]. Streptococcus Equi subspecies Equi represents the most common causal agent for primary bacterial infections of the guttural pouch [2,3,8,10]. The exudate is most frequently unilateral, but involvement of both pouches can sometimes be observed. Radiographic examination can be useful to complement endoscopy for the diagnosis [5]. If the pouch is incompletely filled by exudate, an increased opacity of the ventral part of the pouch can be observed, delineated dorsally by a gas/fluid interface (Figure 4a) [5,11]. This is frequently associated with a mass effect displacing the roof of the pharynx ventrally. If the exudate is too thick, the gas/fluid interface may not be observed, the material can have a convex border, and the area of increased opacity may appear more heterogenous (Figure 4b,c). If the guttural pouch is entirely filled, no residual gas can be observed, and the guttural pouch is materialized as a fluid-filled mass. In cases of chronic disease, chondroids can be observed within the pouch, characterized by the presence of a various number of radio-opaque masses and/or nodules within the pouches (Figure 5) [5,11]. Retropharyngeal adenomegaly can also be associated with guttural pouch empyema and can be detected radiographically by a mass effect caudoventral to the pouch, displacing the caudal border of the pouch cranially (Figure 6) [5]. Ultrasonography is generally not used routinely for the diagnosis of guttural pouch empyema but the presence of a moderate to severe amount of exudate can be detected with ultrasound, which can be useful if endoscopy and radiography cannot be performed (Figure 7) [7]. Ultrasonography can also be helpful in assessing retropharyngeal lymph nodes involvement [4]. CT is also rarely performed in the diagnosis of guttural pouch empyema but can be used in rare cases of a foreign body associated with the empyema [7].

### 3.2. Guttural Pouch Tympany

**Figure 8:**
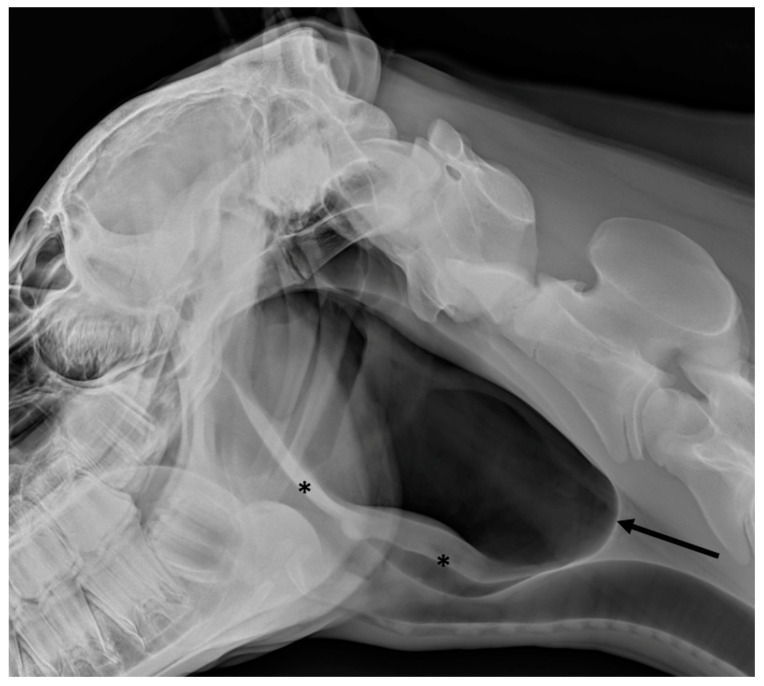
Latero-lateral view of a foal with guttural pouch tympany. The guttural pouch is markedly distended with its caudal border extending caudally up to the level of the caudal endplate of the axis (black arrow). The ventral border of the guttural pouch is displaced ventrally (black asterisks), compressing the airways.

Guttural pouch tympany is a condition of young horses that is a result of trapped air within the guttural pouch due to an excessive amount of tissue at the pharyngeal orifice that allows air to enter the guttural pouch during deglutition but prevents it from exiting [3]. It causes a swelling in the parotid area and can induce respiratory distress (by compression of the nasopharynx) and aspiration pneumonia [3,4,5,11]. Guttural pouch tympany can be easily identified with radiography and is characterized by a severe increase in the guttural pouch size, which can be unilateral or bilateral, extending caudally to the atlas (Figure 8) [5,11]. The caudal border of the dilated pouch appears excessively rounded. The nasopharynx and the larynx can be compressed by the pouch [5]. In some instances, it can be useful to image the thorax if secondary aspiration pneumonia is suspected [5]. Ultrasound and advanced imaging techniques are usually not used to diagnose this condition [7].

### 3.3. Guttural Pouch Hemorrhage

**Figure 9:**
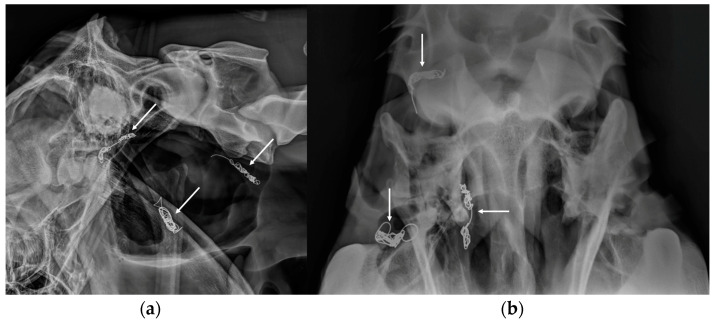
Postoperative latero-lateral (**a**) and ventro-dorsal (**b**) views of a horse after surgical embolization of the internal and external carotid arteries to treat a right-sided guttural pouch mycosis. The position of the metallic coils can be easily identified in the two radiographic views (white arrows).

Bleeding within the guttural pouches is most frequently due to guttural pouch mycosis or injury of the rectus capitis and longus capitis muscles that attach to the skull base [2]. Other rare causes of guttural pouch hemorrhage include foreign bodies, iatrogenic perforation [12] and neoplasia [13]. Diagnostic imaging is usually not performed in the case of guttural pouch mycosis because it does not provide additional information compared to endoscopy, which remains the gold standard for the diagnosis of this condition [2,3]. Nevertheless, fluoroscopy, radiography or ultrasound can be very useful for monitoring the position of the coils used for the surgical treatment of this disease, during surgery or after, for follow-up (Figure 9) [7].

**Figure 10:**
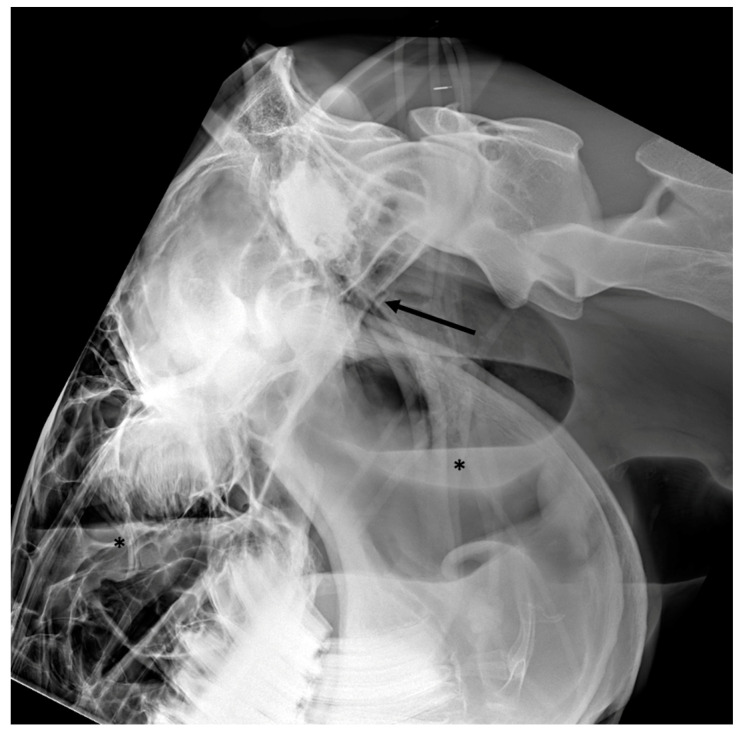
Latero-lateral view of a horse with basilar skull trauma. A bony fragment (black arrow) can be observed caudal to a defect in the basioccipital bone. There is increased and slightly heterogeneous opacity of the longus capitis and rectus capitis muscles. The associated guttural pouch hemorrhage is characterized by homogeneous increased opacity in the ventral part of the guttural pouch (black asterisk) delineated dorsally by a gas/fluid interface. The roof of the pharynx is displaced ventrally.

Basilar skull trauma with injury and avulsion of the rectus capitis and longus capitis muscles occurs when the horse falls over backward [14,15,16]. Such trauma results in avulsion forces exerted by these muscles at their point of attachment to the basisphenoid and basioccipital bones, resulting in fragmentation [14,15,16]. The rectus capitis and longus capitis muscles and the skull base are intimately associated with the medial wall of the guttural pouches and this type of injury results in hemorrhage within the pouches [14,15,16]. This hemorrhage can be detected radiographically by the visualization of a soft tissue opacity in the ventral part of the guttural pouch delineated dorsally by a gas/fluid interface, and a secondary ventral displacement of the roof of the pharynx (Figure 10) [5,14]. A fracture line or irregular outline of the spheno-occipital suture can sometimes be identified. Irregularly shaped avulsion fragments can possibly be observed ventrally to the basisphenoid and basioccipital bones, and sometimes appear close to the stylohyoid bones [5,14,15]. Radiography is limited in its ability to detect and evaluate these fractures because of its poor sensitivity due to complex anatomy of the equine head, superimposition of numerous structures and poor soft tissue differentiation [14,15,16]. Precise evaluation of the configuration of the fracture and any associated traumatic brain injury cannot be achieved with this modality [14,15,16]. If available, CT will give the best evaluation of this complex traumatic injury [14,15,16] by providing the most useful diagnostic information about the type, localization, extension and severity of the basilar skull fracture, and detection of any associated traumatic brain injury.

### 3.4. Temporohyoid Osteoarthropathy

**Figure 11:**
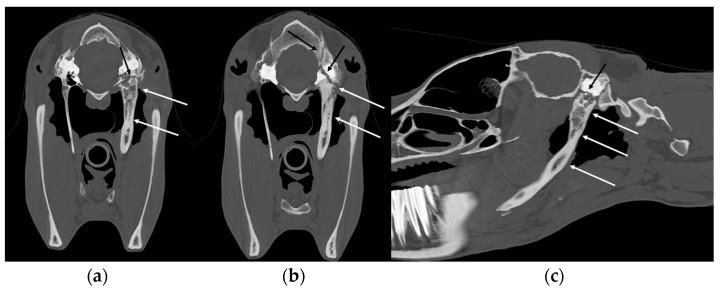
Transverse (**a**,**b**) and sagittal (**c**) CT images (bone window) of a pony with temporohyoid osteoarthropathy. Right is to the left. New bone formation and ankylosis can be observed at the level of the left temporohyoid joint associated with severe thickening of the left stylohyoid bone (white arrows). Several associated fracture lines are present through the left temporal bone (black arrows). Images courtesy of Dr Mickaël Robert.

Temporohyoid osteoarthropathy is a disorder characterized by osseous proliferation of the temporohyoid joint that can lead to neurological disorders, especially facial nerve deficit [17,18,19]. The etiology of this specific condition of horses is poorly understood but it may occur as a sequela to otitis media, upper respiratory infection, or as a primary degenerative disease [9]. This condition leads to progressive new bone formation on the proximal part of the stylohyoid bone, which becomes thicker, and on the petrous part of the temporal bone [9]. An ankylosis of the joint ultimately develops and predisposes to fractures of the temporal and stylohyoid bones [4,19]. The diagnosis is usually performed with endoscopy but diagnostic imaging can be helpful to fully assess any associated osseous damage. Radiographic examination may be useful if a perfectly positioned ventro-dorsal view is performed [6]. An increased opacity of the petrous part of the temporal bone can be observed, centered on the external acoustic meatus and associated with a thickening of the proximal part of the stylohyoid bone [5,17]. Fractures of the stylohyoid bone can sometimes be identified as well [5,17]. CT is more sensitive to detect this condition and allows a more comprehensive evaluation of this region (Figure 11) [9]. Indeed, not only can CT very precisely detect the changes of the temporohyoid joint and stylohyoid bone, but it can also show new bone formation on the ceratohyoid bone, fractures of the temporal bone, fluid filling the tympanic bulla, modeling of the tympanic bulla and resorption of the petrous part of the temporal bone, which cannot be visualized with endoscopy or radiography [8,17,18,19,20]. CT can also allow identification of subclinical bilateral disease in horses with unilateral clinical signs [17].

MRI may also be useful in select cases to diagnose temporohyoid osteoarthropathy [6,9,17], allowing the detection of a fluid signal in the region of the proximal stylohyoid bone and wall of the guttural pouch, structural changes of the cochlea and semicircular canals and fractures in the stylohyoid or petrous temporal bone [9,17]. MRI has a better soft tissue contrast compared to CT and may help to identify inner ear disease or peripheral nerve and brain damage associated with temporohyoid disease [9,17]. Nevertheless, MRI remains less accurate than CT in comprehensively assessing the bony structures of the hyoid apparatus and temporohyoid articulation, and requires general anesthesia which should be avoided if acute vestibular signs are present [3,6,8,9].

### 3.5. Masses Involving the Guttural Pouches

**Figure 12:**
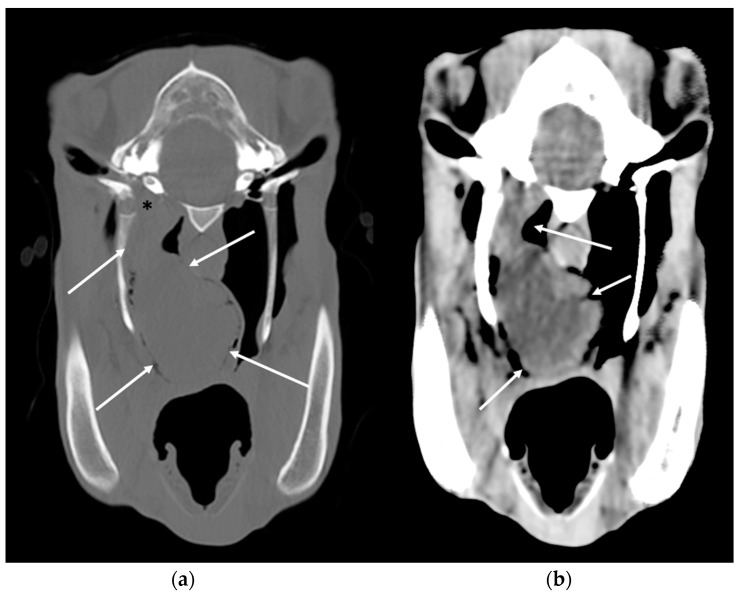
Transverse CT images (bone window (**a**) and soft tissue window (**b**)) of a horse with a squamous cell carcinoma of the right guttural pouch. Right is to the left. A large soft tissue attenuating mass is filling the medial compartment of the right guttural pouch (white arrows) and there is extensive associated lysis of the right tympanic bulla (black asterisk). CT allows a clear understanding of the location and margins of the mass, degree of involvement of the guttural pouch and any associated bony changes. Images courtesy of Dr Mickaël Robert.

The most common cause of a mass effect involving the guttural pouch is adenomegaly of the retropharyngeal lymph nodes secondary to guttural pouch empyema [5]. Hematomas can occur in some cases of trauma, especially to adjacent musculature. Neoplasia involving the guttural pouch is uncommon but cases of fibroma, melanoma, squamous cell carcinoma, hemangiosarcoma and leiomyosarcoma have been described [5,13,21]. A case of a large fungal granuloma secondary to guttural pouch mycosis was also reported [22]. Diagnostic imaging is particularly useful for evaluation of large masses obliterating the pouches because endoscopy may not be possible in these cases and diagnostic imaging provides a better evaluation of the structures surrounding the pouches. Radiographic examination may reveal an increased soft tissue opacity obliterating the air-filled guttural pouch, with a mass effect displacing its contours [5,21]. The contours of the pouch can be compressed if the mass is originating from surrounding structures, or expanded if the mass is located within the pouches [5]. Adenomegaly of the retropharyngeal lymph nodes produces a mass effect at the caudo-ventral aspect of the pouch, pushing the caudal contour of the pouch cranially. The other types of masses can occur anywhere. If the mass is large enough and located lateral or ventral to the guttural pouch, it may be visualized with ultrasound, which can help to identify the origin of the mass and can guide fine-needle aspirations or biopsies. CT provides the best evaluation of the soft tissues and bony structures surrounding the pouches and can be very useful in cases of a mass involving the guttural pouch by giving the best anatomic details that allows us to understand the origin of the mass and its relationship to the guttural pouch, and detect any associated bony lesions (Figure 12) [21]. CT can also be very helpful in precisely determining the margins of the mass, allowing the selection of the most appropriate treatment option [21]. MRI has a very good soft tissue contrast and can be used as an alternative if CT is not available, in order to assess the soft tissue involvement and margins of the mass [9], but it remains less efficient in evaluating the any associated bony lesions and requires general anesthesia.

## 4. Conclusions

Even if endoscopy of the guttural pouch remains the gold standard for identifying most guttural pouch diseases, diagnostic imaging, especially radiographic and CT examinations, and occasionally ultrasound, can be very useful to complement clinical examination and endoscopic evaluation for the diagnosis of some diseases of the guttural pouch in order to precisely determine the prognosis and adapt the treatment.

## Data Availability

Not applicable.
